# Resection of the tumor in the trigone of the lateral ventricle via the contralateral posterior interhemispheric transfalcine transprecuneus approach with multi-modern neurosurgery technology: a case report

**DOI:** 10.3389/fsurg.2024.1371983

**Published:** 2024-06-24

**Authors:** Yunfei Song, Zhen Wang, Jun Zhang, Xiaopeng Cui, Zhuolin Wu, Zilin Zhao, Yan Chen, Suqin Zhang, Xiaowei Zhu, Zhitao Wang, Huijie Zhang, Chao Gao, Shuyuan Yang, Yan Zhao, Xinyu Yang

**Affiliations:** ^1^ Department of Neurosurgery, Tianjin Medical University General Hospital, Tianjin, China; ^2^ Department of Radiology, Tianjin Medical University General Hospital, Tianjin, China; ^3^ Department of Neurosurgery, Tianjin Fifth Central Hospital, Tianjin, China; ^4^ Department of Neurosurgery, Sinopharm Tongmei General Hospital, Tianjin, Shanxi, China; ^5^ Department of Neurosurgery, Yangquan First People’s Hospital, Yangquan, Shanxi, China; ^6^ Department of Neurosurgery, Xi'an No 9 Hospital, Xi'an, Shaanxi, China

**Keywords:** lateral ventricle, trigone, tumor, contralateral, interhemispheric approach, microsurgical resection, surgical technique

## Abstract

Choroid plexus papilloma (CPP) is a rare benign intracranial tumor origin that predominantly manifests in the lateral ventricle in children, accounting for 0.3%–0.6% of all primary intracranial tumors. It is extremely rare to have the CPP in the trigone of the lateral ventricle through the contralateral posterior interhemispheric transfalcine transprecuneus approach (PITTA). Herein, we report this rare case. A 7-year-old girl presented with headache. Magnetic resonance imaging of the brain showed periatrial lesions, and histopathological examination confirmed CPP (WHO grade I). The contralateral PITTA is a safe, effective, reasonable, and appropriate for some lesions in the trigone of the lateral ventricle. It provides a wider surgical angle (especially for the lateral extension) and reduces the risk of disturbance of the optic radiation compared with the conventional approaches. The use of multiple modern neurosurgical techniques, including interventional embolization, intraoperative navigation, microscope, and electrophysiological monitoring, make the procedure much easier and more accurate, and the neuroendoscope adds to the visualization of the microscope and can reduce surgical complications.

## Introduction

Choroid plexus papilloma (CPP) is a rare benign intracranial tumor that arise from choroid plexus epithelium (1). They account for only 0.3%–0.6% of all primary intracranial tumors and just 2%–4% of brain tumors in children (2, 3). The commonest locations in children and adults of CPP is the atrium of the lateral ventricle and the fourth ventricle, respectively (4, 5). Pathological entities situated in this locale commonly manifest with symptoms such as hemorrhage, seizures, visual impairments, and intracranial hypertension (6). Here, we treated a case of CPP arising from the trigone of the lateral ventricle and presenting with typical symptom of headache. The headache disappeared after tumor resection.

Surgical approaches to the periatrial or peritrigonal lesions pose unique neurosurgical challenges because of their proximity to critical structures, including white matter fiber tracts and the overlying cortices (7). The highly functional cerebral cortex and white matter tracts in this region encompass the optic radiations positioned laterally to the ventricle, the supralateral aspect of the postcentral gyrus, and the anteroinferior portion of the thalamus (8, 9). The anterior and posterior choroidal, pericallosal, and splenial arteries supply blood flow to both the surrounding normal parenchyma and lesions within this area. Additionally, crucial deep venous drainage occurs through the internal cerebral veins, vein of Rosenthal, and the straight sinus (10). These structures impose considerable limitations on the surgical corridor to the atrium of the lateral ventricle. Several approaches, like the superior parietooccipital, transtemporal, lateral temporoparietal, posterior transcallosal, and posterior interhemispheric parieto-occipital approaches, have been proposed to safely expos the lesions while addressing the surrounding normal anatomy (7, 8, 10). However, most of these approaches are linked to varying degrees of neurological deficits or limited working corridors (10). There previously used the contralateral posterior interhemispheric transfalcine transprecuneus approach (PITTA) in meningiomas, glioblastoma, and arteriovenous malformation, in the atrium of the lateral ventricle and found that the approach could provide a wider surgical angle and reduce the incidence of complications compared with other conventional approaches (11–13). Thus, it has become a practical surgical approach for lesions in the atrium of the lateral ventricle.

To our knowledge, there are many case reports but no patient in the literature describing the operative detail of the contralateral PITTA for CPP in the trigone of the lateral ventricle. We present the case managed microsurgical via the contralateral PITTA utilizing a combination of multiple modern neurosurgical techniques, including interventional embolization, intraoperative navigation, microscope, neuroendoscopy, and electrophysiological monitoring. We found that the approach could provide a wider surgical angle and reduce the incidence of complications compared with conventional approaches. In this case report, we will describe the associated challenges and advantages of this operative route. The clinical details of this patient will also be presented. The operation was performed by the senior author (X.Y.).

## Clinical presentation

### Patient information

**Figure 1:**
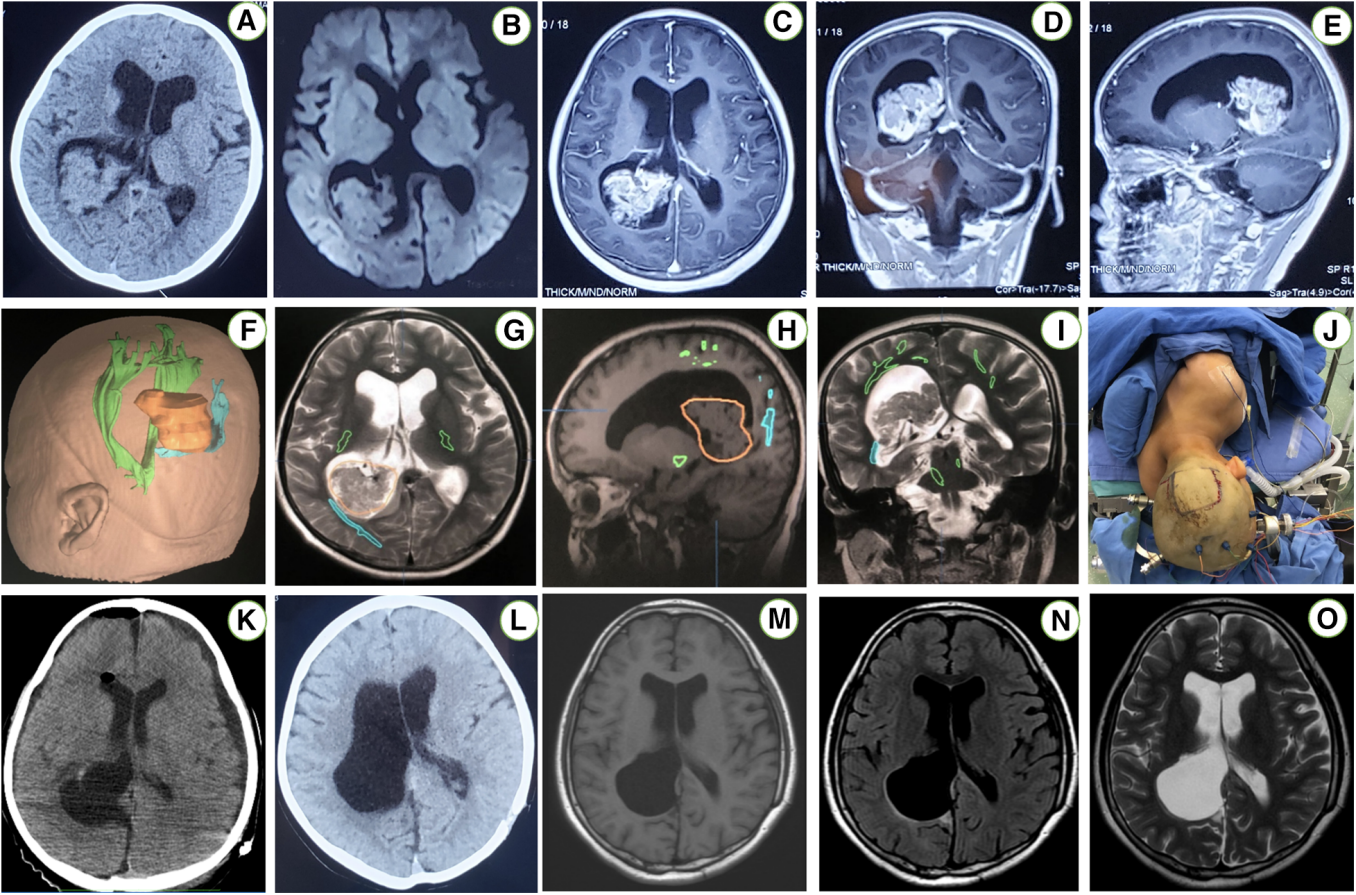
An axial CT scan of the head (**A**) reveals a right periatrial space occupying lesion. An axial T1-weighted MRI without contrast (**B**) demonstrates the exact location of the periatrial tumor. Enhanced images in sagittal, coronal, and transverse views of this patient brain MRI (**C**–**E**) demonstrated berry-like and irregular enhanced lesions in the right trigone of the lateral ventricle. Registration of the image-guided system (**F**–**I**) was performed. The patient position (**J**) in the contralateral PITTA. An axial postoperative CT scan (**K**) reveals a right atrial CPP that was resected via the left PITTA. An axial MRI T1-weighted sequence without contrast (**L**) excludes any unexpected injury to either posterior hemisphere. Postoperative axial T1-flair, T2-flair and T2-weighted without contrast images 4 years later (**M**–**O**) demonstrated complete resection via a contralateral approach.

**Figure 2:**
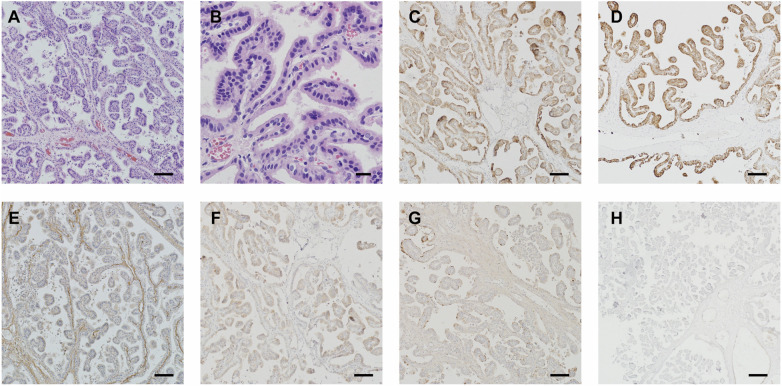
Postoperative pathology: choroid plexus papilloma (WHO I). Immunohistochemical expressions: GFAP (±), EMA (+), keratin (+++), CAM5.2 (+++), P53 (+), syn (++) and olig-2(−). Ki-67LI: 0.6% (**A**–**H**). Scale bar: (**A**,**C**–**H**) 100 μm, (**B**) 20 μm.

A 7-year-old girl was admitted to this hospital because of a 6-week history of headaches and right trigonal tumor. The weight was 40 kg and the body-mass index (BMI) was 23.6. Preoperative imaging demonstrated a right peritrigonal mass suspected to be a CPP (Figures 1A–E). Total resection of this histologically confirmed CPP was evident on Computed tomography (CT) of the head (Figure 1K) 1 day after surgery as well as no injury to the contralateral hemisphere. Postoperative pathology: choroid plexus papilloma (WHO I). Immunohistochemically, expression of GFAP (±), EMA (+), keratin (+++), CAM5.2 (+++), P53 (+), syn (++) was detectable. No immunopositivity was observed for olig-2. Ki-67LI was 0.6% (Figures 2A–H). MRI of the head, performed without the administration of intravenous contrast material, was reportedly negative for any new findings at 4 years of follow-up (Figures 1M–O).

### Operative technique

**Figure 3:**
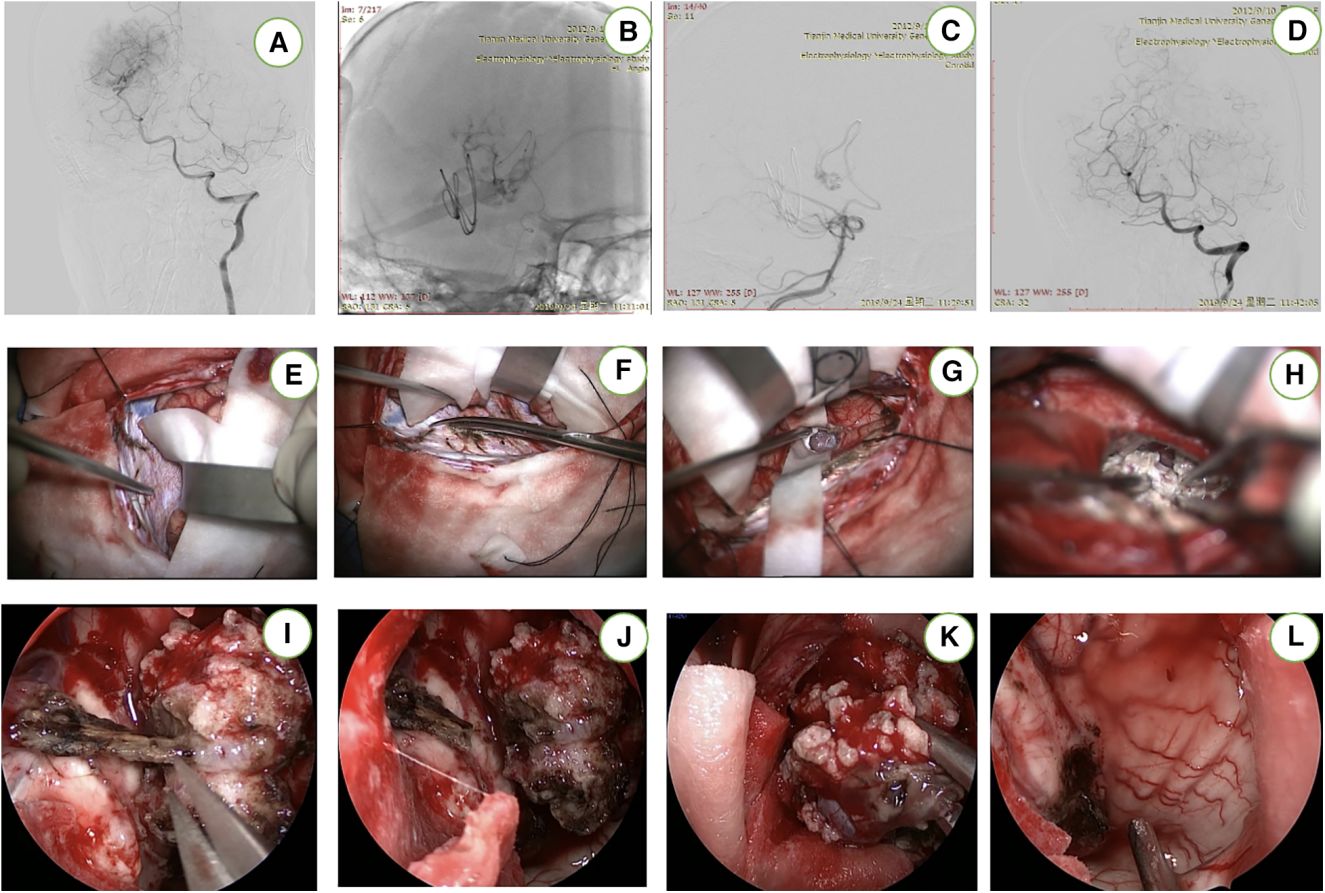
This patient underwent preoperative embolization (**A**–**D**). After cutting the occipital bone, the contralateral precuneus gyrus was located (**E**,**F**). The “T-shaped” incision in the falx has been completed and the tumor was completely resected (**G**,**H**). The endoscope (**I**–**L**) provided supplemental visualization of the temporal horn and the body of the lateral ventricle to check for any residual tumor and to remove the iatrogenic clot.

This patient was under general anesthesia. The endovascular access point was the right femoral artery. After securing access, a microcatheter was advanced into the right PchA for superselective angiography. The right PchA were identified prior to embolization (Figures 3A–D). After embolization, the patient is placed in a three-quarter prone position. The patient's head is rotated to align the axis of the superior sagittal sinus at a 45° angle with the floor. Subsequent to positioning, the image-guided system is registered (Figures 1F–I). The external ventricular drain (EVD) is strategically placed. Throughout the surgical procedure, neurophysiological monitoring is implemented for real-time assessment and guidance.

We employ a diminutive linear incision oriented perpendicular to the superior sagittal sinus, precisely situated over the occipital lobe (Figure 1J). The craniotomy, measuring 4 × 3 cm, is executed on the contralateral parietooccipital region while meticulously monitoring crucial bridging veins within the vicinity. Following the placement of two bur holes over the superior sagittal sinus, a parasagittal craniotomy is meticulously elevated, revealing the entire width and length of the dural sinus. The dura is incised in a horseshoe-shaped manner, aligning with the sinus morphology. Two retraction or tack-up sutures are carefully inserted through the superior portion of the falx, positioned just below the sinus. Microsurgical intervention may be required to release parasagittal bridging veins, enhancing the interhemispheric operative view in the anterior or posterior direction.

**Figure 4:**
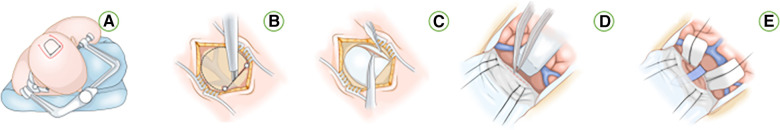
Illustrations and intraoperative photographs of the procedure. (**A**) Patient position and various incision options for approaching a left periatrial lesion while placing the contralateral “approach” hemisphere in the more dependent position to use gravity retraction. (**B**) Bur holes are placed on the superior sagittal sinus and a parasagittal craniotomy is completed while exposing the corresponding segment of the dural sinus. (**C**) A dural opening is made while avoiding the important bridging veins in the region. (**D**) The “T-shaped” incision within the falx cerebri to expose the contralateral medial hemisphere. (**E**) Placement of additional sutures on the falcine dural flaps to increase the transfalcine working angle and corridor to the contralateral atrium.

Approximately 50 ml of CSF is gradually removed through the EVD after dural opening. Cottonoid patties protect the contralateral unaffected hemisphere while a T-shaped incision in the falx is performed as guided by neuronavigation to provide access to the precuneus over the lesion (Figure 1I). The horizontal component of the “T-shaped” incision is made in proximity to the inferior aspect of the superior sagittal sinus, while the vertical segment of the incision is prolonged until the inferior sagittal sinus undergoes coagulation and sectioning (Figures 1J,K). Subsequent to this exposure, the resection of the lesion can proceed through a cortical incision within the precuneus, allowing access to the periatrial region for the removal of the pathological tissue and the exposure of the choroid plexus in the atrium (Figure 1L). Ultimately, the capsule is dissected, and the tumor is completely excised. We employed neuroendoscopy for the evacuation of intraventricular blood clots and comprehensive management of residual tumors or hemorrhage. Subsequently, the falx flap was meticulously closed using continuous sutures, followed by the sequential closure of the dura mater, bone flap, galea, and skin in layers. The (Figures 4A–E) is the Illustrations and intraoperative photographs of the procedure.

Headache was the primary preoperative symptom, and this patient showed disappear after surgery. The operative time was 3 h and 35 min. The estimated blood loss was less than 80 ml; she didn't require intra- or postoperative blood transfusion. Neurological outcomes as scored by the modified Rankin Scale were recorded during the 48 months of follow-up. Good outcomes (modified Rankin Scale scores of 0) were observed in this patient.

## Discussion

The trigone or atrium of the lateral ventricle constitutes a triangular region demarcated by the convergence of the body, temporal, and occipital horns of the ventricle. While relatively infrequent (<1%), it is acknowledged as a distinct site for the occurrence of tumors, including CPPs and meningiomas (14, 15). Additionally, tumors like metastases and gliomas may originate in the peritrigonal region, extending into the atrium (16, 17).

The management of lesions situated in the periatrial region presents a formidable challenge due to their deep location and adjacency to eloquent structures and white matter tracts (7, 18). The determination of the optimal operative approach for this site remains a subject of controversy (19).

In generally, the ideal approach should enable comprehensive microsurgical resection while concurrently mitigating risks to the adjacent structures (20). Kawashima outlined three primary surgical routes: the anterior transsylvian, posterior transcortical/transcallosal, and lateral trans- or subtemporal (21). The anterior transsylvian approach, while providing a narrow corridor suitable for highly vascular lesions, poses a risk to motor fibers and optic radiations (7, 21). The ipsilateral posterior transcortical/transcallosal approach necessitates substantial brain retraction for accessing laterally situated atrial lesions (7, 21). Transtemporal approaches expose optic radiations along the trigone's lateral wall to potential risk (7, 21). An alternative route is the subtemporal corridor traversing the inferior temporal or occipitotemporal gyrus, which is associated with reduced risks of speech and visual field disturbances. However, challenges such as excessive temporal lobe retraction and traction on the vein of Labbe, particularly in the dominant hemisphere, are notable concerns (22, 23).

Alternative operators have supported the utilization of the posterior middle temporal gyrus approach (24). This approach enables prompt accessibility to anterior choroidal arteries and provides effective exposure to tumors that extend into the temporal horn. Nevertheless, the associated risks encompass potential harm to the optic radiations and language impairments in the dominant hemisphere, aphasia, agraphia, alexia, and visual-spatial apraxia (25).

Other surgeons have advocated an ipsilateral parietooccipital interhemispheric approach (24). Visual field impairments varying between 20% and 60% have been documented in association with this methodology. Additionally, Menon et al. identified additional complications, encompassing new motor deficits, seizures, and dysphasia (26). Others have preferred the posterior transcallosal approach (24, 25). The utilization of a contralateral transcallosal trajectory in this approach enables the surgeon to reach lesions with reduced transgression of the cortex and lateral white matter tracts. This approach also mitigates certain deficiencies and risks associated with alternative operative corridors. Nevertheless, the challenges inherent in traversing the corpus callosum for accessing the trigone involve limitations related to a narrow operative corridor and the potential risk of disconnection syndrome (7).

The ipsilateral posterior interhemispheric route is the standard and most commonly used approach for resection of medial periatrial lesions (7). This approach represents the established and frequently employed method for excising medial periatrial lesions. Placement of EVD into the occipital horn serves to deflate the ventricle, reducing hemispheric retraction and facilitating the exposure of the precuneus. However, the PITTA offers a trajectory that is more akin to a “cross-court” orientation towards the lateral aspect of the lesion, concurrently minimizing the extent of hemispheric retraction (7, 23).

One modification to the posterior transcortical/transcallosal approach is the posterior interhemispheric ipsilateral transprecuneus approach (27, 28). While this approach enables the surgeon to avoid optic radiations and the infringement upon the functional temporal cortex, it provides only a narrow operative corridor and necessitates ipsilateral brain retraction (29).

One solution to address the constraints of the narrow operative corridor is the contralateral PITTA (7, 23). This approach involves a modification of the traditional interhemispheric transprecuneus approach by executing a contralateral craniotomy and accessing the ipsilateral precuneus through a transfalcine method (7). By adopting this method, the associated risks of visual and speech deficits linked with the temporal transcortical approach and the potential complications related to somatosensory and visual deficits associated with the transcortical parietooccipital approach are reduced (7). The innovative PITTA route enhances the working angle and avoids excessive retraction of the ipsilateral hemisphere—both crucial elements in minimizing adverse effects and managing vascular lesions within the region (7, 23, 30). We have employed this approach in the treatment of the patient to facilitate the expansion of the operative corridor while mitigating brain transgression.

Several considerations must be carefully addressed to mitigate complications during the preparation of the contralateral PITTA. Particular attention is essential to prevent injury to the straight sinus when executing the “T-shaped” incision within the falx, especially when exposing lesions located more posteriorly. When performing the vertical incision within the falx under the guidance of neuronavigation, it is crucial to execute this incision obliquely from posterior to anterior to protect the straight sinus along the inferior aspect of the vertical falcine incision. This procedural step ensures extensive exposure of the more posterior regions of the precuneus as the falcine flaps are retracted. Following the resection of the lesion, the falcine retraction sutures are removed, and the falcine dural flaps are not sutured together to reconstruct the falx. Emphasis is placed on the pivotal role of neuronavigation in planning the location of the craniotomy flap, ensuring the protection of parasagittal bridging veins, and verifying the operative trajectory. Tractography, integrated with neuronavigation, is employed for navigation around subcortical functional white matter tracts. Subsequently, the removal of blood products and debris is conducted from the temporary EVD.

## Conclusion

In accordance with our empirical observations, the posterior interhemispheric transfalcine transprecuneus approach to trigonal lesions presents itself as a rational, secure, and viable alternative to previously delineated methodologies. This adaptation of the interhemispheric approach mitigates the necessity for substantial ipsilateral cortical resection or retraction, concurrently enhancing the operational angles available to the operator. The exemplified case underscores the proposition that, with judicious patient selection, this alternative trajectory can be judiciously and efficiently applied to a diverse spectrum of pathologies situated within and medial to the trigone of the lateral ventricle.

## Limitation

While this approach demonstrated success in the presented case, less experienced surgeons and even some experienced practitioners may encounter challenges associated with the contralateral midline venous drainage, aberrant deep drainage in the posterior falx, retraction-related injuries to the deep venous system, bilateral visual deficits, and contralateral deep ventricular bleeding without proximal control. Conducting future extensive studies, encompassing similar pathologies, would be imperative to thoroughly explore the potential advantages of the PITTA.

## Data Availability

The datasets presented in this article are not readily available because of ethical and privacy restrictions. Requests to access the datasets should be directed to the corresponding author.
